# Moxibustion Therapy at CV4 Prevents Postoperative Dysuria after Procedure for Prolapse and Hemorrhoids

**DOI:** 10.1155/2013/756095

**Published:** 2013-12-10

**Authors:** Xue-Mei Bian, Ling Lv, Wan-Bing Lin, Hai-Hong Liang, Ying Zhang, Ling-Cong Wang

**Affiliations:** ^1^Department of Anorectal Surgery, The First Affiliated Hospital of Zhejiang Chinese Medical University, No. 54, Youdian Road, Hangzhou 310006, China; ^2^Clinical Evaluation Analysis Center, The First Affiliated Hospital of Zhejiang Chinese Medical University, No. 54, Youdian Road, Hangzhou 310006, China; ^3^Department of Intensive Care Unit, The First Affiliated Hospital of Zhejiang Chinese Medical University, No. 54, Youdian Road, Hangzhou 310006, China

## Abstract

*Objective*. To explore the intervention methods of the patients with dysuria after performing the procedure for prolapse and hemorrhoids (PPH). *Methods*. 100 cases with hemorrhoids were randomly divided into experimental and control groups. The control group received routine nursing care. As comparison, the experimental group, on the basis of conventional care, was treated with moxa roll moxibustion 1 hour after the operation for 30 minutes. The autonomous urination within 1 h, 2 h, 4 h, 6 h, and 8 h after operation and the catheterization rate 8 h after operation of two groups of patients were observed. *Results*. The median time of autonomous urination of control group (8 h) was significantly greater than that of the experimental group (6 h) (*P* < 0.001). Cox regression analysis showed that the moxibustion therapy was positively correlated with automatic micturition in the patients after PPH. The probability of automatic micturition in the experimental group was 2.032 times that in the control group (RR = 2.032, 95% CI: 1.278~3.230). The catheterization rate of control group (38%) was significantly higher than that of the experimental group (10%) (*P* < 0.001). *Conclusion*. The Guanyuan acupoint moxibustion can prevent dysuria after PPH and reduce the urethral catheterization.

## 1. Introduction

Longo [1], an Italian scholar, first reported the procedure for prolapse and hemorrhoids (PPH) in 1998. Since then, PPH has been widely applied worldwide because it is consistent with anatomical physiology, is simple to perform, and provides little postoperative pain and a rapid recovery. Dysuria is a common complication after operations involving the anus, with urinary retention representing a severe manifestation [2–5]. Patients who suffer from dysuria also easily experience irritability of symptoms, which in turn may worsen dysuria. Therefore, the development of preventive therapies and early interventions in postoperative dysuria are critical; in traditional Chinese medicine, the “preventive treatment of diseases” has these same goals.

Based on this background, the authors conducted the present study between May 2011 and May 2013. One hundred patients who underwent PPH and satisfied the inclusion criteria were randomized into 2 groups and were both given routine nursing care. In addition, the experimental group was treated with moxibustion at CV4 1 h after PPH, which yielded satisfactory outcomes. The detailed operation and results are given below.

## 2. Clinical Data and Methods

### 2.1. Clinical Data

One hundred patients with mixed hemorrhoids, admitted to the Department of Anus and Intestine Surgery in our hospital between May 2011 and May 2013, were randomized into control and treatment groups using the SPSS17.0 statistical program. Patients who were diagnosed with mixed hemorrhoids and were given PPH after continuous epidural anesthesia were included. Patients with primary diseases that could induce urinary retention before surgery, those who had a history of obstructive uroschesis, such as prostatoplasia, and those who did not follow the study protocol were excluded. Informed consent was obtained from each patient before surgery.

### 2.2. Methods

Patients in the control group were given urination training in bed, with men using urinals and women using adult diapers. Before the surgery, bladder emptying was conducted. Continuous epidural anesthesia was performed by the same anesthetist, and the operator was the director of the Department of Anus and Intestine Surgery. An HYG-34 Anastomat (HJZ34, Haida Medical Equipment Co., Ltd., Changzhou, Jiangsu Province, China) was used, and 500 mL of Ringer's solution was infused during the operation. The visual analog scale was applied to control the score of perianal pain to no more than “2”. During the perioperative period, uniform treatment was performed according to the doctor's advice, including anti-inflammatory therapy (metronidazole and sodium chloride injection 100 mL, i.v. g.t.t., b.i.d.; NS 100 mL; and cefuroxime sodium for injection 1.5 g, i.v. g.t.t., b.i.d., 3 days for a course) and hemostasis therapy (NS 250 mL plus haemocoagulase Agkistrodon for injection 2.0 g, i.v. g.t.t., q.d.). The transfusion of metronidazole and sodium chloride injection 100 mL was controlled within 1 h, cefuroxime sodium for injection 1.5 g within 1 h, and haemocoagulase Agkistrodon for injection 2.0 g within 2 h.

Based on the treatment performed in the control group, the treatment group was additionally given moxibustion at CV4. A pure moxa roll was used (Xuyu Pharmaceutical Factory of Traditional Chinese Drug, Jiangsu, China); this moxa roll was composed of wormwood fiber, was cylindrical and ring-girdling, and measured 20-21 cm in length and 1.9–2.1 cm in diameter (SFDA approval number Z32020153; patent number: ZL201020297603.X).

The moxibustion therapy was performed as follows. One hour after the surgery, the patient was asked to lie on his or her back on the bed with the abdominal skin exposed. CV4 was chosen, located 3 cm below the umbilicus along the abdominal anterior median line, approximately equal to 4 horizontal fingers (the overall width of the forefinger, middle finger, ring finger, and little finger). The moxa roll was lit and put in a Fan's moxibustion firepot (trademark: Xin'aixing; patent number: ZL200920079158.7). The firepot was fixed on the abdomen of the patient. The moxa roll was completely burned and then cooled off, which took a total of about 30 min. During the procedure, the physician frequently made his rounds, examining the skin near the site of moxibustion. Urethral catheterization was performed in patients who did not urinate 8 h after PPH.

### 2.3. Data Recording

Automatic micturition and urethral catheterization in the 2 groups were recorded at 1, 2, 4, 8, and beyond 8 h after the surgery.

### 2.4. Statistical Analysis

SPSS17.0 was used for statistical analysis. Measurement data were expressed as means ± standard deviations or medians, while enumeration data were expressed as rates or percentages (%). Independent-samples *t* tests were used for comparisons between the 2 groups, while chi square tests were applied for rate comparisons. Log-rank tests were used for survival analysis using comparisons of automatic micturition rates, while Cox regression was used for exploring the factors influencing automatic micturition in patients after PPH.

## 3. Results

### 3.1. Patient Recruitment Flowchart

**Figure 1:**
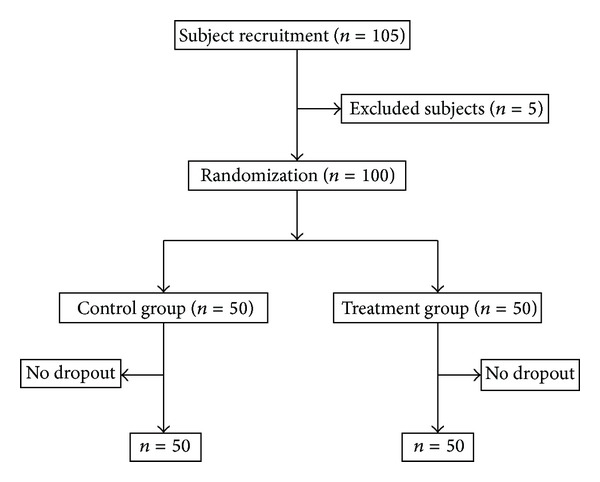
Flowchart of the randomized, controlled trial.

A total of 105 patients were recruited, among whom 5 were excluded due to combined prostatoplasia (Figure 1).

### 3.2. Baseline Data

Baseline data including gender, age, reason for surgery, and medical history did not differ between the 2 groups (*P* > 0.05; Table 1).

### 3.3. Survival Analysis

**Figure 2:**
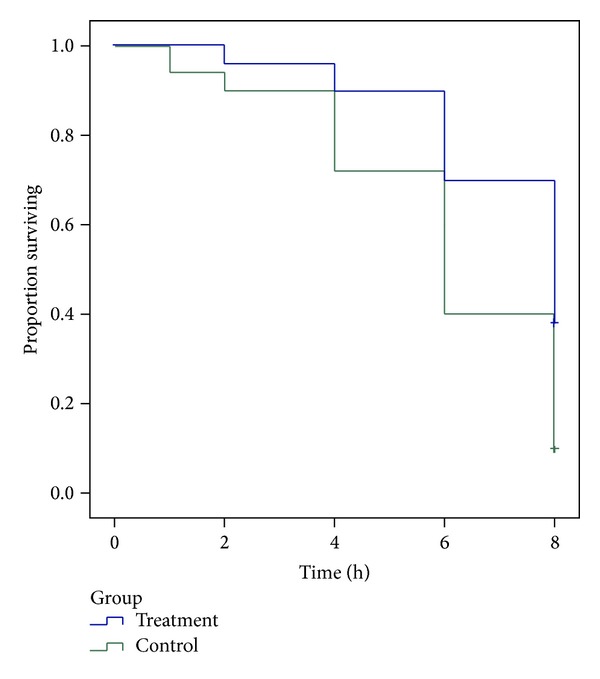
Survival analysis.

Whether to appear autonomous urination was regarded as the primary endpoint (status = 1 expressed autonomous urination, while status = 0 expressed the absence of automatic micturition and urethral catheterization was required). The time at which automatic micturition presented was taken as the survival time, and automatic micturition times were compared between the 2 groups (Table 2 and Figure 2).

As shown in Table 2, the median time of the presence of automatic micturition was 8.00 h in the control group and 6.00 h in the treatment group. Thus, automatic micturition occurred significantly earlier in the treatment group than in the control group (*P* < 0.001).

### 3.4. Cox Regression Analysis

Cox regression analysis was used for exploring the factors influencing automatic micturition in patients after PPH. Status and time were taken as dependent variables, while age, sex, and group were taken as independent variables. The data revealed that moxibustion therapy was positively correlated with automatic micturition in postoperative patients after PPH. The probability of automatic micturition in the treatment group was 2.032 times that in the control group (RR = 2.032, *P* = 0.003, 95% CI: 1.278–3.230), suggesting that the intervention of moxibustion therapy was the key factor influencing the time of automatic micturition in postoperative patients after PPH (Table 3).

### 3.5. Comparison of the Rate of Urethral Catheterization between the 2 Groups

The rate of urethral catheterization in the control group was 38.00%, while that in the treatment group was significantly lower (10.00%, *P* = 0.001; Table 4).

## 4. Discussion

Urinary retention (UR), the most common postoperative complication after PPH, is defined by an inability to effectively empty the bladder on spontaneous voiding within 8 h after PPH, with bladder urine volume being greater than 600 mL, or ability to effectively empty the bladder on spontaneous voiding, with residual urine volume being greater than 100 mL [6]. The rate of UR after PPH is 1.5%–16.7% [2–5]. Some studies have demonstrated that the rate of UR in lumbar plexus anesthesia (0%) was significantly lower than that in epidural anesthesia (40%) [7]. In our hospital, we primarily used epidural anesthesia and innervation of both the anus and bladder arises from the same spinal segment (S2). Epidural anesthesia can cause loss of anal sensory function and anal sphincter loosening and can also anesthetize pudendal nerves, which would block the micturition reflex of the primary centrum and interfere with physiological micturition. In addition, after the anus is stuffed with Latin sponge and gauze following PPH and a T bandage is used at the perianal region for hemostasis by compression, reflex spasms of the sphincter vesicae can result. Postoperative perineum pain and fear of pain on defecation can also cause UR. In order to exclude diet or rapid infusion, which can cause premature filling of the bladder, the patients in this study were asked to fast for 8 h before the surgery, with water deprivation for 4 h. Fluid infusion was conducted uniformly with 500 mL during the surgery and 650 mL after the surgery. Six hours after PPH, the patients could drink water, thereby allowing patients to avoid leptochymia.

In clinical practice, UR is often treated by means of mental nursing, induction of urination, acupuncture, neostigmine acupoint injection, and catheterization. Induction of urination is commonly used, and methods for this include listening to flowing water, local hot compression, rinsing, and massage; however, these methods are not quite satisfactory due to complicated operations and slow onset. Acupuncture at SP6, SP9, and CV3 has satisfactory effects, but needle insertion and acupoint selection are difficult for clinical practitioners. Neostigmine acupoint injection has better efficacy in micturition, but this procedure may be considered invasive since patients may feel pain. Neostigmine acupoint injection also has contraindications, such as occlusive ileus, angina, bronchial asthma, and urinary obstruction. Catheterization is the most direct and effective method for UR but is also the most direct cause of nosocomial infection.

CV4 [8] is located on the Bladder Meridian of Foot-Taiyang. As is described in *Zhenjiu Zisheng Jing*, Guanyuan acupoint (CV4) can treat most diseases related to failure of urination. CV4 is the crossing acupoint of the conception vessel and the 3 *Yin* meridians. Moxibustion at CV4 can regulate San Jiao, warm and invigorate kidney qi, and promote bladder gasification to improve urination and defecation. Wormwood [9] belongs to the feverfew family of plants and is bitter in flavor, warm in nature, and fragrant in odor. It is flammable and produces moderate heat when burned. When used for moxibustion, it can warm and regulate meridians and collaterals, activate qi, promote blood circulation, eliminate dampness and cold, relieve swelling, and recuperate depleted yang. It is also applicable for disease prevention and health preservation. Studies have demonstrated that acupuncture at CV4 and other acupoints is effective for relieving UR [10, 11]. Moxibustion therapy at CV4 is noninvasive, does not require exposure to patients' private body regions, and has satisfactory safety and compliance. In this present study, moxibustion therapy was conducted 1 h after PPH, the duration required for patients to return to the ward, nurses to relay care instructions, postoperative doctors' orders to be filled, and moxibustion procedures to be prepared. This was the earliest time that postoperative moxibustion therapy could be performed, and this improved the time until automatic micturition and relieved patient pain.

Making patients safe and comfortable has been the subject of intense research interest. The prevention and early intervention of dysuria have also attracted more attention. Early prevention of dysuria is a goal of the “preventive treatment of diseases” in traditional Chinese medicine. In this study, moxibustion was performed 1 h after PPH for 30 min, using Fan's firepot fixed on the abdomen. The procedure was simple, and a nurse frequently examined the patient during routine rounds while moxibustion therapy was performed, evaluating the skin near the site of moxibustion. No skin scalding was observed for any of the patients. The data revealed that the time and rate of automatic micturition in the treatment group were both significantly superior to those in the control group, suggesting that moxibustion was the key factor influencing postoperative automatic micturition after PPH.

One limitation of the present study was that blinding could not be conducted. Additionally, the “placebo” effect of wormwood could not be avoided, and, therefore, operation bias could also not be avoided.

In conclusion, moxibustion at CV4 can prevent dysuria after PPH and reduce the need for catheterization.

## Figures and Tables

**Table 1:** Baseline data of the 2 groups.

	Control group *N* = 50	Treatment group *N* = 50
Age (years), mean ± SD	37.00 ± 7.09	37.30 ± 7.25
Female, no. (%)	21 (42)	24 (48)
Male, no. (%)	29 (58)	26 (52)
Reason for surgery		
Mixed hemorrhoid	50	50
Hypertrophy of anal papilla	6	7
History of cholecystitis	2	2
History of hypertension	2	2
History of fracture	2	1
History of knee joint surgery	1	2
Gastric ulcer	2	2
History of blood transfusion	2	1

**Table 2:** Distribution of spontaneous urination times in PPH patients after the 2 treatments.

Group	*N*	Urination time (h)						Median time (h)	log-rank test	
		1	2	4	6	8	8^+^		χ^2^	P
Treatment	50	3	2	9	16	15	5	6.00	13.992	<0.001
Control	50	0	2	3	10	11	24	8.00		

Total	100	3	4	12	26	26	29	8.00		

**Table 3:** Results of Cox regression analysis.

Variable	*B*	SE	*χ* ^2^	*P*	RR	RR 95% CI	
						Lower	Upper
Moxibustion	0.709	0.236	8.995	0.003	2.032	1.278	3.230
Sex	−0.226	0.239	0.891	0.345	0.798	0.500	1.275
Age	0.012	0.016	0.585	0.444	1.012	0.981	1.045

Assignment: moxibustion: yes = 1, no = 0; sex: male = 1, female = 2.

**Table 4:** Comparison of spontaneous urination rates between the 2 groups.

Group	Spontaneous urination		Nonspontaneous urination		*χ* ^2^	*P*
	*N*	Percentage (%)	*N*	Percentage (%)		
Treatment	45	90.00	5	10.00	10.746	0.001
Control	31	62.00	19	38.00		

Total	76	76.00	24	24.00		
